# Cough of Remote Origin

**Published:** 1883-06

**Authors:** W. E. Casselberry

**Affiliations:** Physician to the Throat and Chest Department of the South Side Dispensary, Chicago Medical College; 70 Monroe St., Chicago


### ARTICLE II.

### COUGH OF REMOTE ORIGIN. BY W. E. CASSELBERRY, M. D.,

Physician to the Throat and Chest Department of the South Side Dispensary, Chicago Medical College.

Involuntary cough is a reflex phenomenon, produced by irritation of certain peripheral ends, branches, or trunks of the pneumogastric nerve. The impression conveyed to the cough center, said to be located at the point of origin of the pneumogastric nerves, in the medulla oblongata, produces therein a reaction, which manifests itself by the spasmodic action so well known as cough—a full inspiration, closure of the glottis, and forcible expulsion of the pent up air under contraction of the expiratory muscles and diaphragm.

The several localities in the distribution of the par vagum from which cough can originate, for clinical consideration, may be classed in two divisions, viz., those within, and those without the respiratory mucous tract.

With the first division, which for contradistinction, we may designate as coughs of proximal origin, we are not specially interested in this paper, farther than may serve to elucidate our subject proper. To this end, we will mention the "cough spots" of Stoerk.* This highly original Viennese professor has experimentally demonstrated, under laryngoscopic observation, that there are certain "spots" in the respiratory tract, any irritation of which will, with certainty, cause cough ; namely, the inter-arytenoid fold, supplied by the internal branch of the nervous laryngeus superior, the posterior fibrous walls of the larynx and trachea, and the bifurcation of the trachea, supplied through branches of the nervus vagus. These observations are confirmed by Lennox Browne, of London.† From a more more clinical standpoint, Stoerk‡ includes the bronchi and bronchioles as cough areas ; but from both experimental and clinical observations, excludes the lung parenchyma, disease of which, he maintains, does not cause cough unless accompanied by bronchial congestion, irritation or inflammation.

The second division of the subject includes coughs originating without the respiratory tract—those in which the exciting cause is more remotely located—in the alimentary canal, heart, medulla oblongata, etc. The present is an age of instrumental precision in medical science, and we are prone to overlook the more intangible ætiological influences. The once common "stomach cough" is now a hackneyed diagnostic phrase, relegated to the

*Klinik der Krankheiten des Kehlkopf der Nase und des Rachens, S. 220.
†Diseases of the Throat.
‡Op. cit.

ranks of the superannuated, quack and unprofessional. At its mere utterance the scientific physician (especially the younger generation) involuntarily shrugs his shoulders, proceeds to use his laryngoscope and auscult the patient. We see only with our mirror a laryngitis or tracheitis and hear the evidence of a bronchitis or more serious lung trouble.

A recent case has brought this subject to our attention, connected with which are points we wish to suggest thought upon rather than hope to entirely elucidate.

Mr. A., an active business man in the prime of life, had complained of cough for a year or more, better at times and again worse, apt to occur in severe paroxysms, but usually annoying him also in a more continuous form. He had had an occasional tickling sensation, but complained more of a sore, raw feeling in the larynx. During this time he had suffered constantly from dyspepsia with uncomfortable sensations or actual pain after eating, acid eructations, occasional vomiting, constipation and anorexia. A paroxysm of cough was at times terminated by an attack of vomiting. He had undergone various treatments, had consumed quantities of cough mixtures, homœopathic pellets, and proprietary remedies.

We saw the patient first in Oct., 1882. He was somewhat emaciated, pale, and nervous about himself, laid particular stress upon his cough, and disregarded his dyspeptic symptoms as a matter of little consequence. Laryngeal examination disclosed a slight reddish yellow discoloration of the vocal cords, and redness of the arytenoid cartilages, indications merely of a light chronic laryngitis. The trachea was somewhat congested. There was considerable follicular pharyngitis with congestion of the anterior pillars of the fauces, and slight thickening of the submucous connective tissue at the angles of the pharynx, behind the pharyngo-palatine folds. Physical examination of the chest gave no evidence of bronchitis or disease of the lungs.

The treatment consisted of a careful regulation of the diet, an occasional laxative, and 1-20 grain strychnia sulphate before each meal. Inhalation from the atomizer of tannic acid solution twice daily, occasional intralaryngeal applications of nitrate of silver solution, 20 grains to the fluid ounce, and local treatment for the follicular pharyngitis. The patient improved rapidly, gained much in weight and appearance, the cough disappearing *pari passu* with the improvement in the dyspeptic symptoms, both cough and dyspepsia having vanished at the end of six weeks. At this time the laryngoscopic appearances, however, were practically the same as when first seen. The arytenoid cartilages were, perhaps, not so red, but the cords remained unchanged. Treatment had benefited the follicular pharyngitis, some of the larger follicular eminences having been destroyed by means of the London-paste, and others by nitrate of silver. The patient at this time is still well, but by advice continued to regulate his diet carefully and take occasionally some strychnia pellets, which act also by keeping his bowels in proper order. Meeting him a few days since in a street car, he remarked: "Doctor, my throat continues to behave itself beautifully." And upon query in reference to his dyspepsia, he remarked, that that too was all right.

This case we regarded from the first as one of reflex dyspeptic cough, or "stomach cough," if we may be permitted to use the expression. The duration and severity of the cough were out of all proportion to the small amount of laryngeal inflammation present, and its severity appeared to go *pari passu* with the severity of the dyspeptic symptoms. Its paroxysmal character, also, was too marked for a purely laryngeal cough, and the cessation of a paroxysm upon an attack of vomiting, was very significant. Moreover, the patient's impaired nutrition, in the absence of any chest symptoms, indicated his digestive organs as the chief cause of trouble. True, there was some chronic inflammation of the larynx, but apart from the fact that three-fourths of the people living in certain climates and seasons suffer from slight laryngeal catarrh, the *cough* in this case was sufficient to *produce* irritation, and the sensation of soreness and rawness complained of, I presume to have been muscular and directly caused by the cough. Finally the entire subsidence of the cough with the very slight change in the larynx, agrees as to the correctness of the diagnosis.

Acid eructations will aggravate or cause pharyngeal irritation, which may extend thence into the larynx, especially to the arytenoid cartilages and interarytenoid fold ; or these portions of the larynx which form the anterior boundary of the pharynx as it merges into the œsophagus, may be affected by direct contact with the eructated acid material. Such an action was entirely probable, to some extent, in the case related, as the arytenoids were the only laryngeal portions which improved. In view of this same interarytenoid fold being one of the "cough spots" of Stoerk, its irritation in this way might be said to explain the entire gastric nature of the case, and any nervous influence be denied. Such an exclusive view of the case we do not believe, however, to be justified by an analysis of the symptoms.

The English-writing specialists have little or nothing to say relative to the subject, but *apropos* we will quote from Stoerk :*

"Through the experiments of Mayer and Pribraur,† supported by the observations of Traube and Henoch, the question of the so-called stomach cough, has appeared in a new phase. The older school of medicine, which looks upon disturbances in the digestive tract, especially collections of gas in the stomach and intestines, as etiological factors in many pathological states of the respiratory organs, has, in true presentiment, recognized a certain association between affections of the stomach and cough thus appearing reflexly, and has designated this cough as stomach cough. We must admire these observations, rendered certain, as they are, by more recent experiment. It is a known and much observed fact, that many violent and long enduring paroxysms of cough end with vomiting and find their termination through the same. One can assume now that the cough is caused by irritation of the terminal ends of the pneu. mogastric nerve in the stomach, exactly as it happens from certain 'spots' in the respiratory tract."

It is possible for a dyspepsia to supervene upon a pharyngitis, that is, the sequence of events is reversed, a primary pharyngeal catarrh, producing reflexly gastric disturbance. But this is apt to occur only in the most advanced stages of pharyngeal and naso-pharyngeal catarrh.

---

* Klinik der Krankheiten des Kehlkopfs der Nase und des Rachens, § 223.

† Sitzungsberichte der K. Academie der Wissensch B. 66 Abath. 3.

The stomach, although one of the most common, is not the only remote source of cough. We would use this instance but as a text for the whole subject.

Diseases of the naso-pharynx and pharynx, goitre, aneurism of the aorta, innominata and carotids. Cardiac affections, cervical or mediastinal lymphadenitis, œsophageal disease, in fact, disease of any organ involving one or more branches of the pneumogastric nerve.

Charcot mentions cough and dyspnœa among the symptoms of compression of the upper part of the spinal cord (Stoerk),* possibly due to involvement of the cough center in the medulla oblongata.

Aurists frequently observe cough arising from irritation of the inferior wall of the external auditory canal and of the pharyngeal opening of the eustachian tube—in the former case to be attributed to involvement of the auricular branch of the pneumogastric, in the latter case to the pharyngeal branches and plexus.

Cohen† and Robinson‡ both remark, that impairment of the voice and cough may occur in follicular pharyngitis without any visible implication of the larynx, "apparently by extension of the nervous influence of the pneumogastric nerve." This is frequently observed with public speakers. Enlarged tonsils, too, are a prolific source of cough in children. Goitre and other cervical and mediastinal tumors occasion cough, which is probably at times remotely reflex, from pressure or other involvement of the pneumogastric trunk or its branches. In accordance with a general physiological law, reflex cough is, however, more easily excited by irritation of peripheral nerve ends. Nevertheless, numerous German experimentors (Stoerk§) have succeeded in exciting cough by irritation of the trunkal portions of the pneumogastrics.

Carotid aneurism can act in the same way—as a tumor. The nervus laryngeus recurrens being exclusively motor in function

* Op. cit.

† Diseases of the Throat and Nasal Organs, p. 182.

‡ Nasal Catarrh, p. 132

§ Op. cit.

and therefore composed entirely of centrifugal fibers, it is decidedly improbable that its compression by aneurism of the arch of the aorta, or of the right subclavian, could produce a reflex cough, although cough is a most common symptom in these cases. Still it is not to be understood that in *all* cases of aneurism and of cervical and mediastinal tumors the cough is remotely reflex, for it may be due to direct compression of the trachea or a main bronchus, in the former case easily demonstrable by the laryngoscope, and in the latter by physical examination. Compression near the bifurcation would irritate the "cough-spot" at that point, and the cough be thus explained.

Functional and organic cardiac derangements are frequently accompanied by troublesome cough, but how much of purely nervous influence is concerned in these cases, would be difficult to say. As pointed out by Dr. Beverly Robinson in a paper read before the American Laryngological Association,* congestion of the larynx or entire respiratory tract with cough and other symptoms is not an uncommon result of debilitated or deranged cardiac action. Chronic interstitial nephritis, cirrhosis of the liver, spleen, etc., likewise by rendering the heart *relatively* weak, bring on congestive cough.

Thus we see occasioned another and partially distinct class of coughs, which, including in the same category cough produced by pressure upon the trachea or bronchi, may be said to arise directly from a proximal cause and yet be traceable to a remote origin.

As regards diagnosis little need be said. That little, however, must be said in testimony of the efficacy of the laryngoscope. Its value in the precise diagnosis of laryngeal and tracheal diseases is well known, and its use among practical physicians rapidly becoming more prevalent. In the case just related, it served as a valuable aid to diagnosis by exclusion, demonstrating the absence of sufficient cause for cough in the larynx and trachea. To this end its use is almost a necessity in all coughs of remote origin. To the routine practitioner a cough is a cough to be met by the inevitable cough-mixture; whilst the physician

* Archiv. of Laryngology, vol. III. NO. 3.

of an "investigating turn of mind," he who looks his patient all over before prescribing, will easily discover the *fons et origo* of the symptom and treat accordingly.

70 MONROE ST., CHICAGO.

